# A Multilingual Digital Microlearning Intervention for Oral Health in Refugee Shelters: Randomized Controlled Trial

**DOI:** 10.2196/95562

**Published:** 2026-06-15

**Authors:** Maxi Katharina Müller, Fabian Schulz, George Jogho, Simone Steffens, Kirstin Vach, Ahmad Amro Baradee, Daniel R Reissmann, Benedikt C Spies, Anna-Lena Hillebrecht

**Affiliations:** 1 Faculty of Medicine, Department of Operative Dentistry and Periodontology, Medical Center—University of Freiburg, Freiburg, Germany, Freiburg, Germany; 2 Department of Prosthetic Dentistry, Faculty of Medicine, Medical Center—University of Freiburg, Hugstetter Str. 55, Freiburg, 79106, Germany, 49 761 270-48440; 3 Section of Health Care Research and Rehabilitation Research, Institute of Medical Biometry and Statistics, Medical Faculty and Medical Center - University of Freiburg, Germany, Freiburg, Germany; 4 RefuDocs Freiburg e. V, Wirthstr. 7AFreiburg, 79110, Germany; 5 Institute of Medical Biometry and Statistics, Medical Faculty and Medical Center - University of Freiburg, Germany, Freiburg, Germany; 6 Center for Data Science, Eberswalde University for Sustainable Development, , Eberswalde, Germany; 7 Department of Prosthodontics and Materials Science, Centre for Dental Medicine, Medical Centre, University of Leipzig, Leipzig, Germany, Leipzig, Germany

**Keywords:** refugees, digital health, health equity, health literacy, oral health literacy, microlearning, randomized controlled trial

## Abstract

**Background:**

Refugees frequently face language and access barriers to preventive oral health information. Brief multilingual digital interventions may help reduce such barriers in shelter settings.

**Objective:**

This randomized controlled trial evaluated whether a multilingual digital microlearning video improved plaque control and selected self-reported oral health–related behaviors among adults living in refugee shelters.

**Methods:**

A 2-arm, parallel-group randomized controlled trial was conducted among 86 adults living in 2 municipal refugee shelters in Germany. Participants were randomized (1:1) to receive either a multilingual 4-minute oral hygiene microlearning video or delayed access (control group). Plaque index and gingival index were assessed clinically at baseline and at 2-month follow-up. Secondary outcomes included questionnaire-based measures of oral health literacy–related cognitions and self-reported oral health behaviors. Between-group differences in change scores were analyzed using 2-sided tests; exploratory multivariable regression analyses were conducted to assess potential effect modifiers.

**Results:**

Follow-up was completed by 83 (97%) of 86 participants. Plaque index decreased more in the intervention group than in the control group (mean change −0.21, SD 0.27 vs mean change −0.04, SD 0.17; *P*=.002). Gingival index decreased in both groups, but the between-group difference was not significant. Among questionnaire-based outcomes, toothbrushing frequency increased substantially, whereas the remaining oral health literacy–related items showed small numerical changes that did not reach statistical significance or remained stable. Approximately three-quarters of participants in the intervention group (32/42, 76%) reported reviewing the video at least once.

**Conclusions:**

Brief multilingual digital microlearning improved plaque control and self-reported toothbrushing frequency in refugee shelters. Effects on broader oral health literacy–related outcomes were limited and should be interpreted cautiously. Larger, prospectively powered trials with longer follow-up periods and blinded outcome assessment are warranted.

## Introduction

Digital access to health information is not dependent on staff or time, is cost-effective, and can thus be made available to more individuals more promptly and with fewer barriers. Digital health information has expanded rapidly over the past decade, yet vulnerable and displaced populations continue to face profound informational inequities. Refugees often encounter linguistic, structural, and administrative barriers that limit access to preventive services and reliable health education. These information barriers contribute to avoidable disease burden and widen existing health disparities. Scalable, low-threshold digital interventions may offer a pragmatic approach to reducing such inequities, particularly in high-mobility and resource-constrained settings [1-3].

The global number of forcibly displaced people increased from approximately 65 million in 2015 to more than 82 million by the end of 2020 and has continued to rise in recent years, reaching more than 120 million worldwide [4,5]. Oral health is a key but often overlooked dimension of refugee health, with untreated dental diseases reducing quality of life and increasing demands on public health care systems [6,7]. Poor oral health can also be linked to systemic conditions such as cardiovascular disease and diabetes, and it contributes to psychosocial stress, stigma, and reduced employability [8]. These interrelations underline the importance of addressing oral health as part of overall refugee health.

In Germany, oral health has improved over recent decades, yet refugees remain an underserved population [9]. During the initial period after arrival, dental care is generally restricted under the Asylum Seekers Benefits Act to treatment required for acute illness or pain. In addition to uncertainties regarding insurance status and repeated transfers, this can lead to most carious lesions remaining untreated, with substantial consequences for individual well-being and increased health care costs [10]. Administrative access models during the initial reception period may further contribute to inequities in health care use, with voucher-based systems associated with less equitable access than electronic health card models or regular access [11]. Similar disparities have been reported across other European host countries, including Greece, Sweden, and the United Kingdom, where refugees show higher caries prevalence, lower dental service use, and greater unmet treatment needs than the resident populations [12].

Disparities are compounded by conditions in countries of origin, where oral health systems often have fewer dental professionals, limited preventive services, and a higher prevalence of caries [13-17]. In host countries, barriers such as low income, limited education, language difficulties, and differing cultural health beliefs further undermine oral health behaviors [18].

Digital and video-based approaches have shown promise for oral health promotion, particularly when adapted to the needs of populations facing language or literacy barriers [19-21]. Web-based oral health promotion programs have also been shown to improve knowledge, attitudes, self-efficacy, and oral hygiene practices in adult populations, supporting the potential of digital formats for preventive oral health education [22]. A mixed methods systematic review of digital health literacy interventions for forced migrant populations found that 83% of included studies reported positive outcomes despite cultural, linguistic, and practical barriers [23]. Recent research has shown that adults with migration experience demonstrate significantly poorer oral health and lower oral health literacy than the general population, with oral health literacy emerging as an independent predictor of clinical outcomes [24]. Educational interventions targeting refugees have also demonstrated improvements in oral health literacy, awareness, and self-reported oral hygiene behaviors following structured oral health education programs [25]. In parallel, evidence from the development of a digital prevention program indicated that tailored mobile health interventions can successfully improve oral health literacy and oral hygiene in these groups, although such approaches are often complex and resource intensive [26]. Existing oral health promotion resources are often designed for majority-language populations and may require sustained engagement with mobile apps or web-based platforms, stable internet access, and literacy levels that cannot be assumed in newly arrived displaced populations. In addition, many available tools lack cultural and linguistic tailoring to the heterogeneous contexts of refugee shelters and may therefore fail to reach those with the greatest preventive needs. These limitations informed the development of GlobeSmile as an ultrabrief, multilingual, low-threshold microlearning intervention designed for access via standard smartphones using QR codes and requiring minimal digital skills or prior knowledge.

The “GlobeSmile” intervention was designed as a highly accessible, low-threshold tool to provide refugees with essential oral health information in their own languages. The short video focused on simple, practical instructions for daily oral hygiene procedures, such as toothbrushing technique, the use of fluoride, and preventive practices, in line with the clinical practice recommendations of the *Fédération Dentaire Internationale* (commonly known as FDI World Dental Federation) for maintaining good oral health and with evidence-informed international consensus recommendations on toothbrushing and oral hygiene behaviors [27,28]. Therefore, this study aimed to evaluate the effectiveness of the GlobeSmile video intervention among refugees in Germany. It was hypothesized that participants receiving the intervention would show greater improvements in plaque index (PI) and gingival index (GI), as well as in selected oral health literacy outcomes, than participants in the control group.

## Methods

### Trial Design

This study was designed as a 2-arm, parallel-group, superiority randomized controlled trial with individual randomization and a 1:1 allocation ratio. The trial was conducted in 2 municipal refugee shelters in Freiburg, Germany, between November 2023 and February 2024. Outcomes and assessment time points were defined a priori, and no protocol modifications were made after trial initiation.

The study was approved by the ethics committee of the University of Freiburg prior to participant enrollment. Due to administrative delays, registration in the German Clinical Trials Register (DRKS00032017) was completed after recruitment and follow-up had been completed; therefore, the trial is classified as retrospectively registered. No changes were made after study initiation to the primary objective, primary end point, eligibility criteria, intervention, allocation procedure, or core study design.

### Participants

Eligible participants were adults (aged ≥18 years) residing in the participating shelters who were able to understand at least 1 of the languages in which the intervention was available (Arabic, Kurdish, Persian [*Dari* and *Farsi*], or Ukrainian) and had at least 10 teeth per jaw to allow reliable clinical index assessment. Individuals with acute dental emergencies or those requiring antibiotic prophylaxis were excluded. All eligible residents present during the recruitment period were invited to participate. A total of 86 participants met the inclusion criteria, provided written informed consent, and were enrolled. Participants with clinically relevant findings during examination received individual treatment recommendations and, if necessary, were referred to the university dental clinic to avoid delays in necessary care.

### Randomization and Allocation

Randomization was performed after baseline assessment using a stratified allocation procedure to balance groups by age and native language. The allocation sequence was generated independently by the statistician (KV), who was not involved in recruitment, intervention delivery, or clinical outcome assessment. Allocation was concealed from the examining dentist until baseline assessments had been completed.

### Intervention

The intervention was developed through an iterative participatory process involving dental professionals, translators, and individuals with refugee experience recruited through collaborating shelter networks and community contacts. Development was informed by oral health literacy principles emphasizing plain language, visual demonstration, multilingual narration, and accessibility across different literacy levels. Draft scripts and visual materials underwent repeated refinement based on feedback regarding comprehensibility and usability within refugee shelter settings.

The GlobeSmile intervention [29] is publicly accessible online via YouTube (Alphabet Inc) and consists of a short, multilingual educational video providing simple instructions on daily oral hygiene practices. The full script is provided in Multimedia Appendix 1.

The educational content focuses on key preventive behaviors, including toothbrushing technique, the use of fluoride-containing toothpaste, brushing duration and sequence, tongue cleaning, and rinsing, and was informed by international oral hygiene recommendations [27,28]. The video has a total length of approximately 4 minutes and combines simple animations with multilingual voice-over explanations to ensure accessibility across different literacy levels. The final version was made available in Arabic, Kurdish, *Dari*, *Farsi*, and Ukrainian. The control group received access to the video after completion of the follow-up to ensure ethical equipoise.

### Implementation and Outcomes

The primary outcomes were changes in the PI (according to Silness and Loe [30]) and GI (according to Loe and Silness [31]). Secondary outcomes were assessed using a study-specific multilingual questionnaire developed for this field setting, addressing oral hygiene behaviors as well as oral health literacy–related cognitions.

Follow-up was scheduled approximately 2 months after baseline. This interval was chosen pragmatically, as longer retention in communal shelter settings could not be assumed, while still allowing sufficient time for the adoption of oral hygiene behaviors and the detection of short-term changes in plaque accumulation and gingival inflammation.

Clinical examinations were conducted at baseline and follow-up under standardized field conditions. At both time points, the PI and GI were assessed.

All examinations were conducted by a single trained and calibrated examiner (FS) using portable dental equipment and headlamps to ensure comparable examination conditions across time points. Prior to data collection, the examiner underwent calibration with an experienced practitioner to ensure measurement reliability (interrater reliability: PI Cohen κ >0.8 and GI Cohen κ >0.8).

Caries experience (Decayed, Missing, and Filled Teeth index according to Klein and Palmer [32]) was recorded at baseline as a descriptive characteristic.

Participants in the intervention group viewed the video on a tablet computer immediately after the baseline clinical examination in one of the available intervention languages that they reported understanding. They also received a printed QR code for continued access, allowing them to rewatch the video as often as desired during the 2-month study period (Figures S1 and S2 in Multimedia Appendix 1). Participants in the control group received no intervention during the trial but were provided with access to the video via a QR code after completion of the follow-up examination.

Questionnaires were administered in the participants’ native languages at baseline and follow-up, with assistance from trained translators to ensure comprehension and cultural appropriateness. The survey included items on demographic information (age, sex, country of origin, native language, and education); refugee experience; oral health knowledge, attitudes, and perceived self-efficacy; oral hygiene behavior (brushing frequency and utensils); nutrition (according to Woelber et al [33]); physical activity; and tobacco and alcohol use (Tables S1 and S2 in Multimedia Appendix 1). The questionnaire was developed specifically for this multilingual field study and included behavioral, lifestyle-related, and oral health literacy–related items informed by oral health behavior literature, anti-inflammatory dietary recommendations, and the Internal-External Locus of Control Short Scale–4. Translation was supported by trained translators and reviewed for comprehensibility and cultural appropriateness. The questionnaire was pilot-tested informally prior to implementation and was not intended as a formally validated oral health literacy instrument.

### Blinding

Participant blinding was not feasible due to the nature of the intervention. At follow-up, clinical assessments were performed before participants were asked about intervention use, such that the examiner was not routinely aware of group allocation. However, due to the shared shelter setting, complete assessor blinding could not be guaranteed. Examinations were conducted by a single calibrated examiner under standardized conditions, and data analysis was performed using coded group labels.

### Sample Size

No formal a priori sample size calculation was performed; the sample size was determined pragmatically by the number of eligible shelter residents who were present during the recruitment period and willing to participate. All individuals who met the inclusion criteria and provided written informed consent were enrolled. On the basis of the observed variability of the PI, which represented the primary outcome expected to change most rapidly, 40 participants per group were sufficient to detect moderate between-group differences in PI change with 90% power at a 2-sided significance level of α=.05.

### Statistical Analysis

Continuous outcomes are reported as mean (SD) to aid interpretability. For univariate analyses, within-group changes in PI and GI were assessed using Wilcoxon signed-rank tests. Between-group comparisons of change scores were conducted using 2-sided *t* tests as the primary contrasts. Multivariable linear regression models were used to examine associations of age, sex, smoking status, and group allocation with changes in PI and GI and additionally served as sensitivity analyses for the nonparametric results. Analyses were performed in Stata (version 19; StataCorp), with a significance level of α=.05.

### Ethical Considerations

Ethics approval was obtained from the Ethics Committee of the University Medical Center Freiburg (23‐1392-S1) prior to participant enrollment. All participants provided written informed consent before participation. Study information and consent materials were provided in relevant participant languages, with translator support where needed to ensure comprehension. Participant data were pseudonymized at the time of collection and analyzed in deidentified form to ensure confidentiality and data protection. No financial compensation was provided. Participants with clinically relevant findings during examination were informed and referred for appropriate dental care.

## Results

### Participant Flow and Retention

**Figure 1.**
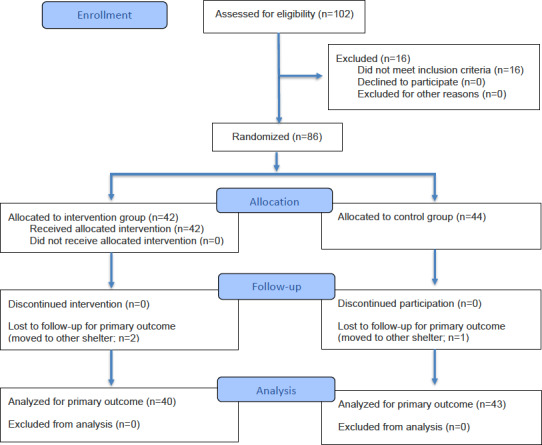
CONSORT (Consolidated Standards of Reporting Trials) flow diagram of participant recruitment, randomization, allocation, follow-up, and analysis in a 2-arm parallel randomized controlled trial evaluating a multilingual oral health microlearning intervention among adults living in 2 municipal refugee shelters in Freiburg, Germany, between November 2023 and February 2024.

A total of 86 residents of the 2 participating shelters were assessed for eligibility, all of whom met the inclusion criteria, consented, and were enrolled in the study. Of these, 42 (48.8%) participants were randomized to the intervention group and 51.2% (44/86) to the control group. Follow-up at 2 months was completed by 83 (97%) participants. In total, 3.5% (3/86) participants were lost to follow-up (2/42, 4.8%) in the intervention group and 1 (2.3%) in the control group. No missing clinical or questionnaire data were observed among participants who completed follow-up (Figure 1).

### Baseline Characteristics

Baseline sociodemographic and clinical characteristics were broadly comparable between groups (Table 1). Participants had a mean age of 35.4 (SD 12.6) years, and 54.7% (47/86) of participants were female. The largest subgroups originated from Ukraine, Iraq, and Afghanistan, with native languages reflecting this distribution. All participants reported sufficient understanding of at least 1 of the intervention languages.

Educational level, lifestyle factors, and baseline clinical indices (PI; GI; and the Decayed, Missing, and Filled Teeth index) showed no relevant differences between groups.

**Table 1.** Baseline sociodemographic, behavioral, and clinical characteristics of participants randomized to the intervention or delayed-access control group in a randomized controlled trial conducted in refugee shelters in Freiburg, Germany.

Characteristics	Intervention group (n=42)	Control group (n=44)	Total participants (n=86)
Age (years), mean (SD; range)	36.0 (12.9; 18-67)	34.8 (12.4; 18-60)	35.4 (12.6; 18-67)
School education (years), mean (SD; range)	8.5 (1.9; 3-11)	8.4 (2.5; 3-12)	8.4 (2.2; 3-12)
Time since displacement (years), mean (SD; range)	6.7 (5.5; 0.2-16.0)	6.9 (5.9; 0.1-16.0)	6.8 (5.7; 0.1-16.0)
Sex, n (%)			
Male	18 (42.9)	21 (47.7)	39 (45.3)
Female	24 (57.1)	23 (52.3)	47 (54.7)
Country of origin, n (%)			
Ukraine	14 (33.3)	14 (31.8)	28 (32.6)
Iraq	11 (26.2)	11 (25)	22 (25.6)
Afghanistan	6 (14.3)	6 (13.6)	12 (14)
Kosovo	5 (11.9)	4 (9.1)	9 (10.5)
Nigeria	2 (4.8)	4 (9.1)	6 (7)
Georgia	2 (4.8)	3 (6.8)	5 (5.8)
Serbia	2 (4.8)	0 (0)	2 (2.3)
Macedonia	0 (0.0)	2 (4.5)	2 (2.3)
Native language, n (%)			
Ukrainian	14 (33.3)	14 (31.8)	28 (32.6)
Kurdish	11 (26.2)	11 (25)	22 (25.6)
Persian (*Dari* and *Farsi*)	6 (14.3)	6 (13.6)	12 (14)
Others	11 (26.2)	13 (29.5)	24 (27.9)
Oral health behaviors, n (%)			
Toothbrushing (≥2 times per day)	13 (31)	15 (34.1)	28 (32.6)
Use of interdental cleaning aids	15 (35.7)	16 (36.4)	31 (36)
Current smoker, n (%)	19 (45.2)	22 (50.0)	41 (47.7)

### Primary Clinical Outcomes

Mean PI score decreased from 1.21 (SD 0.48) to 1.01 (SD 0.46) in the intervention group (mean change −0.21, SD 0.27; *P*<.001), whereas the control group showed only a small change (mean change −0.04, SD 0.17; *P*=.23). The between-group difference in PI change was significant (*P*=.002).

GI decreased in both groups (intervention group: mean change −0.11, SD 0.23; *P*=.002 and control group: mean change −0.07, SD 0.16; *P*=.006), but the between-group difference was not significant (*P*=.55). Table 2 summarizes all primary outcomes.

**Table 2.** Clinical oral health outcomes at baseline and 2-month follow-up among intervention and control participants in a randomized controlled trial of a multilingual oral health microlearning intervention^a^.

Clinical outcome	Intervention group				Control group				Between-group *P* value
	T_0_^b^	T_1_^c^	Change (T_1_–T_0_)	*P* value	T_0_	T_1_	Change (T_1_–T_0_)	*P* value	
Plaque index, mean (SD)	1.21 (0.48)	1.01 (0.46)	−0.21 (0.27)	<.001	1.12 (0.39)	1.08 (0.34)	−0.04 (0.17)	.23	.002
Gingival index, mean (SD)	0.49 (0.49)	0.37 (0.44)	−0.11 (0.23)	.002	0.38 (0.35)	0.30 (0.29)	−0.07 (0.16)	.006	.55

a No significant differences between the sexes could be identified for plaque index or gingival index (Figure 2).

b T_0_: baseline assessment.

c T_1_: follow-up assessment.

**Figure 2.**
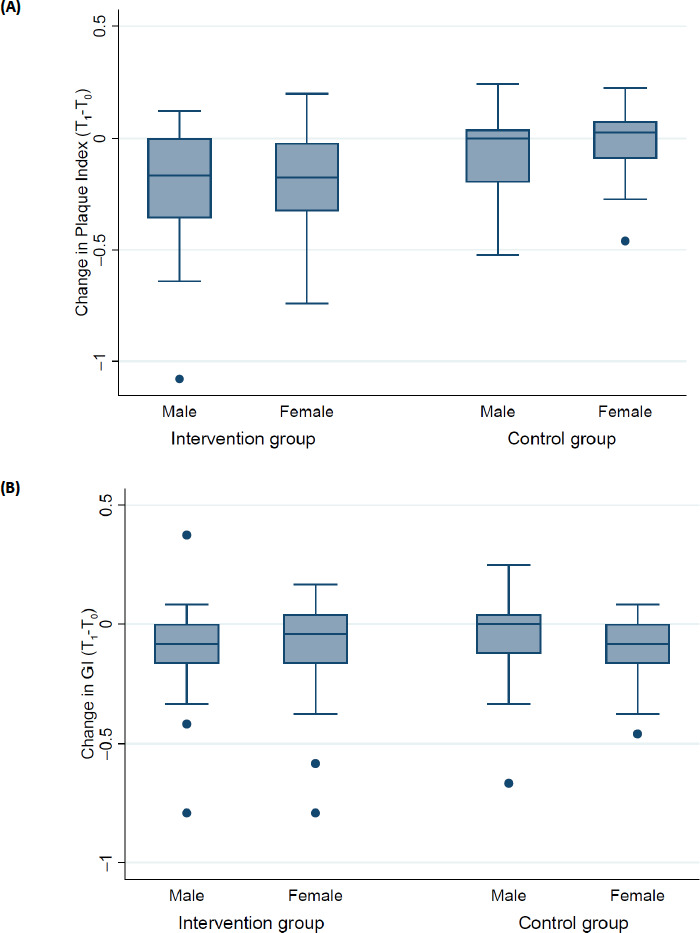
Distribution of individual changes from baseline (T_0_) to follow-up (T_1_) in (**A**) plaque index and (**B**) gingival index, stratified by study group and sex among adults living in refugee shelters. Boxes indicate IQRs, horizontal lines indicate medians, whiskers indicate ranges excluding outliers, and dots represent individual observations.

### Secondary Outcomes: Oral Health Literacy Components

Questionnaire findings were mixed (Multimedia Appendix 1). Brushing frequency improved significantly, while selected cognitive items showed favorable but nonsignificant trends. Other items remained largely unchanged.

### Additional Analyses and Effect Modifiers

Exploratory adjusted regression analyses were conducted to assess whether participant characteristics were associated with changes in clinical outcomes. In these models, group allocation remained significantly associated with PI change, confirming greater plaque reduction in the intervention group (β=0.1662, 95% CI 0.0697-0.2626; *P*=.001). No significant intervention effect was observed for GI change (β=0.0361, 95% CI −0.0504 to 0.1226; *P*=.41).

Age was significantly associated with greater improvements in both clinical outcomes. With each additional year of age, participants showed slightly greater reductions in PI (β=−0.0046, 95% CI −0.0087 to −0.0006; *P*=.02) and GI (β=−0.0042, 95% CI −0.0078 to −0.0005; *P*=.02). Sex and smoking status were not significantly associated with changes in PI or GI. These findings were consistent with the primary analyses and supported by nonparametric sensitivity analyses (Multimedia Appendix 1).

### Intervention Adherence and Digital Engagement

Reported engagement with the video was generally high: 76.2% (32/42) of participants in the intervention group rewatched the video via the QR code at least once during the study period. Participants who reported repeated viewing tended to show larger reductions in PI, suggesting that repeated exposure to the content may be beneficial.

## Discussion

### Principal Findings

This randomized controlled trial evaluated whether a brief microlearning video could improve oral hygiene outcomes and oral health literacy among refugees living in communal shelters in Germany. The findings indicate that a simple digital intervention may improve plaque control and selected cognitive determinants of oral health behavior, even in settings with limited access to preventive care and substantial linguistic diversity. Oral health served as a pragmatic model for addressing health information barriers in an underserved population.

### Interpretation of Findings

The intervention was associated with a greater reduction in PI than that observed in the control condition, suggesting improved oral hygiene performance independent of brushing frequency. In addition to a general Hawthorne effect, the baseline dental examination itself may have acted as a behavioral prompt, increasing oral hygiene awareness in both groups. The script recommends a brushing duration of approximately 3 minutes, which is slightly longer than commonly cited minimum recommendations. This was intentionally chosen as a behavioral strategy to encourage sufficiently thorough cleaning, acknowledging that actual brushing duration in everyday practice is often shorter than recommended. Video-based educational interventions have previously been shown to influence health behaviors across a range of contexts by facilitating observational learning and modeling of desired behaviors, supporting the plausibility of such mechanisms in this study [34]. One possible interpretation is that the intervention affected brushing technique rather than brushing frequency. Toothbrushing is often a habitual and partly automatic behavior, and previously learned routines may interfere with the acquisition of new oral hygiene skills [35]. Because PI and GI involve examiner judgment, the absence of fully blinded outcome assessment may have influenced estimates of effect size and should be considered when interpreting the findings. However, the examiner was not routinely aware of group allocation during follow-up assessments, which may have reduced the risk of observer bias.

Questionnaire findings were mixed; only brushing frequency improved significantly, while selected cognitive items showed favorable but nonsignificant trends. These findings suggest that broader oral health literacy–related effects should be interpreted cautiously. This interpretation is consistent with recent meta-analytic evidence indicating that psychologically informed interventions can improve oral hygiene behaviors, plaque levels, and self-efficacy in the short term by targeting cognitive and motivational determinants of oral health behavior [36]. These findings are consistent with established health literacy frameworks, in which increased knowledge, enhanced confidence, and a stronger internal health locus of control are key mechanisms linking educational interventions to improvements in oral health behaviors and clinical outcomes [18,24,34]. Therefore, the data support a plausible mechanism whereby culturally adapted microlearning enhances both oral health literacy and the application of brushing techniques, leading to reduced plaque accumulation.

Participants demonstrated high digital engagement. Three-quarters of the participants (32/42, 76.2%) rewatched the video at least once, and individuals who reported repeated viewing tended to show larger PI reductions. Although the frequency of repeated viewing was insufficient to support a formal dose–response analysis, the observed pattern suggests that repeated exposure may reinforce skill acquisition and message internalization. This finding is consistent with behavior change theory and supports the potential value of microdose repetition in digital health literacy interventions [23,36]. The study thereby highlights an important practical implication: even minimal recurrency of exposure may meaningfully enhance effectiveness.

### Comparison With Existing Research

The results complement current international evidence documenting pronounced oral health inequities among refugees and asylum seekers, driven by structural access barriers, linguistic limitations, inconsistent entitlements to care, and fragmented service pathways [6,10,12,17]. Consistent with reports from Germany and other European countries, participants exhibited high baseline levels of untreated caries and low oral health literacy, factors strongly associated with reduced service use and poorer health outcomes [10,18,24].

Digital health interventions have been proposed as a promising strategy to reduce inequities in forced migrant populations, particularly where conventional education is difficult to deliver due to low literacy, language heterogeneity, or limited access to health professionals. Similarly, behavioral interventions using visual cues and motivational messaging have demonstrated improvements in plaque control among schoolchildren with refugee and immigrant backgrounds, highlighting the potential of simple visual communication strategies for promoting oral self-care in underserved populations [37]. These findings are consistent with previous work suggesting that digital health literacy interventions can be beneficial in forced migrant populations [23]. These findings add experimental evidence to this literature and suggest that ultralow-threshold, multilingual microlearning tools can produce clinical improvements without requiring complex infrastructure or advanced digital skills.

Compared with multicomponent mobile health programs that demand extensive engagement, device access, and continuous coaching [26], the simplicity of the present intervention may offer greater scalability for settings such as shelters, reception centers, or transit facilities. Importantly, however, digital education alone cannot compensate for structural constraints, including the emergency-focused care entitlements that limit preventive dental treatment for refugees in Germany [10]. Therefore, the intervention should be viewed as a complementary public health strategy rather than a substitute for system-level reform.

The observed effects may in part reflect reinforcement of previously known oral health information rather than entirely novel knowledge acquisition. However, this distinction is less relevant from a public health perspective: even when basic knowledge exists, accessible, linguistically tailored microlearning may facilitate translation into daily practice and measurable clinical outcomes. Therefore, in populations facing structural and informational barriers, reinforcement and accessibility may represent key mechanisms of effective prevention.

### Strengths and Limitations

Strengths of this study include its randomized controlled design, the use of calibrated clinical assessments, the high follow-up rate of 97%, and the naturalistic implementation within refugee shelters, which enhances ecological validity. The participatory development of the video, involving translators and individuals with refugee experience, likely contributed to cultural acceptability and comprehension.

Several limitations should be considered. First, the sample size was modest and determined pragmatically rather than through a formal a priori power calculation. Therefore, the study was primarily exploratory and may have been underpowered to detect smaller between-group differences, particularly for secondary outcomes and subgroup analyses. Second, the study population was heterogeneous with regard to country of origin, language, migration history, and social circumstances. Although this heterogeneity reflects the real-world composition of refugee shelters and strengthens the practical relevance of the findings, it may also have increased variability in responses to the intervention, limited the precision of effect estimates, and reduced the interpretability of subgroup patterns. Third, the 2-month follow-up period restricts conclusions regarding long-term behavioral change and the sustainability of effects. Fourth, some degree of contamination between groups cannot be entirely ruled out, given the shared living environment. Fifth, gingival outcomes are difficult to influence over a short period without professional prophylaxis, which may partly explain the absence of a significant between-group effect for GI. Complete assessor blinding could not be guaranteed, and some risk of observer bias remains. Questionnaire outcomes were assessed using a pragmatic study-specific instrument rather than a formally validated oral health literacy scale, which limits precision, reliability, and comparability with other studies. Finally, adherence was assessed by self-report, and objective use metrics for repeated video viewing were not available.

### Implications for Public Health and Policy

This trial provides actionable evidence supporting the implementation of multilingual digital microlearning as part of preventive strategies for displaced populations. Although evaluated here in the context of oral health, the intervention model may be transferable to other preventive health domains in which multilingual, low-threshold access to information is limited. The intervention was feasible to deliver, well accepted, and associated with a meaningful improvement in plaque control and limited favorable changes in selected self-reported outcomes. Because it is low cost, language adaptable, and accessible via QR code, it could be readily integrated into refugee shelters, reception centers, community health programs, and nongovernmental organization–led services.

From a health equity perspective, the results indicate that well-designed tools can reduce information barriers, thereby supporting the overarching aims of Inclusion Health, which focuses on improving health outcomes and access to care for socially excluded populations. Nevertheless, digital education should be embedded within broader multisectoral approaches that address restricted care entitlements, financial and administrative barriers, and limited access to preventive dental services for refugees [6,10,18]. Without structural reforms, improvements in knowledge and technique may not translate into long-term disease reduction. Because the intervention requires minimal infrastructure and can be distributed via QR codes or standard video platforms, it represents a highly scalable digital public health strategy that could be adapted to other preventive health domains and multilingual populations.

Detailed information on previous dental attendance and prior exposure to oral health education interventions was not systematically collected. These factors may have influenced baseline behaviors and responsiveness to the intervention. Future trials should assess prior dental care use and previous preventive education exposure as potential moderators of intervention effects.

### Future Research

This study illustrates how even ultrabrief digital interventions can produce measurable clinical change when delivered in linguistically adapted formats. The model may be transferable to other preventive domains where information barriers limit health equity. The GlobeSmile video was developed without external funding and relied on deliberately simplified, self-produced visual materials to ensure rapid and low-cost implementation. Although this didactic reduction enhances accessibility and scalability, future iterations could explore more professionally designed or visually enriched formats to assess whether enhanced aesthetic and pedagogical features further increase engagement and effectiveness. Future studies should evaluate long-term outcomes, assess cost-effectiveness, and determine whether combining video-based education with in-person demonstrations or support from community health workers provides additional benefits. Research across diverse refugee settings and language groups could assess generalizability and inform implementation strategies. Additionally, investigating the mechanisms linking improved oral health literacy to clinical change could support theory-based optimization of digital microlearning interventions.

### Conclusions

Brief multilingual digital microlearning may represent a feasible and scalable strategy to improve plaque control and reduce health information barriers in refugee shelter settings. Larger, prospectively powered trials with longer follow-up periods are warranted.

## Supplementary material

10.2196/95562 Multimedia Appendix 1 Supplementary materials related to the GlobeSmile multilingual oral health micro-learning intervention, including the intervention video script, oral health questionnaire, and outcome measures, QR-code access card used for participant access to the intervention.

10.2196/95562 Checklist 1 CONSORT checklist.

## Abbreviations

GI: gingival index
PI: plaque index
