# Supplementary material for: A Multilingual Digital Microlearning Intervention for Oral Health in Refugee Shelters: Randomized Controlled Trial
Source: J Med Internet Res. 2026 Jun 15;28:e95562. doi: 10.2196/95562 (PMC13268633; doi:10.2196/95562)

**Supplementary Material:**

**Supplementary Material S1: Video-Script**

*„Teeth become diseased when food residues and bacteria adhere to the tooth surface for too long. This not only does not look nice, but can also lead to pain and bad breath. In the worst case, the teeth are lost.*

*With a toothbrush and toothpaste, food residues and bacteria on the teeth can be removed, so that the teeth remain not only white, but also healthy. To keep the teeth healthy, it is important to clean all tooth surfaces. Here are our tips:*

*Rinse the toothbrush with water, then apply toothpaste to the toothbrush. It is good if there is fluoride in the toothpaste, it strengthens the teeth. If there is no other way, toothpaste without fluoride is also okay. It is important that all tooth surfaces are brushed.*

*Clean the outer surfaces of the teeth from red to white. This means that you start with the toothbrush on the red gum and then brush over the white tooth. Do this with a light pressure. If the gums bleed, this may be a sign that you need to brush this area more efficiently. Bacteria damage not only the teeth but also the gums.*

*The inner surfaces of the teeth must also be cleaned. Here, too, it is best to brush from red to white. A little toothpaste foam can sometimes squirt out of the mouth...*

*You may scrub the chewing surfaces properly...to the front and back...the bacteria and food residues adhere particularly well to the small hills and ditches....*

*Finally, the tongue is also treated, which may feel a little unusual at first...But bacteria also adhere to the surface of the tongue and must be removed.*

*Finally, rinse well with water and spit out all the bacteria and food residues that were previously in your mouth.*

*In total, brushing all tooth surfaces and the tongue should take about 3 minutes. If you do this in the morning after breakfast and in the evening before sleeping, your teeth should not only stay healthy, but also beautifully white.“*

**Supplementary Material S2: Oral Health Questionnaire**

**Supplementary Table 1:** Lifestyle habits

|  | daily | several times a week | once a month | never |
| --- | --- | --- | --- | --- |
| 1. smoking tobacco |  |  |  |  |
| 2. alcohol |  |  |  |  |
| 3. drug use |  |  |  |  |
| 4. chew betel leaf |  |  |  |  |
| NUTRITION | | | | |
| 5. carbohydrate-rich food /  flour products |  |  |  |  |
| 6. sweets, juices |  |  |  |  |
| 7. fruits, vegetables |  |  |  |  |
| 8. fish |  |  |  |  |
| 9. coffee |  |  |  |  |
| 10. tea |  |  |  |  |
| 11. sugary drinks |  |  |  |  |
| PHYSICAL ACTIVITY | | | | |
| 12. walks |  |  |  |  |
| 13. sports |  |  |  |  |

*Questions about lifestyle habits using a 4-point scale; “NUTRITION” in reference to anti-inflammatory dietary recommendations according to Wölber et al. [20]

**Supplementary Table 2:** Cognitive and Behavioural Outcomes Related to Oral Health Literacy

|  |  | Doesn’t apply at all | Doesn’t really apply | Undecided | Applies | Completely true |
| --- | --- | --- | --- | --- | --- | --- |
| 1. | I have control over my oral health. |  |  |  |  |  |
| 2. | If I practice conscientious oral care, my teeth stay healthy. |  |  |  |  |  |
| 3. | My oral health depends largely on factors that I cannot control myself. |  |  |  |  |  |
| 4. | My oral health is determined by fate. |  |  |  |  |  |
| ADDITIONAL QUESTIONS | | | | | | |
|  |  | Doesn’t apply at all | Doesn’t really apply | Undecided | Applies | Completely true |
| 5. | I should brush my teeth more often, but I don’t. |  |  |  |  |  |
| 6. | I know what I should brush my teeth with. |  |  |  |  |  |
|  |  | Not at all | Every two days | Once a day | Twice a day | Three times a day |
| 7. | I brush my teeth.. |  |  |  |  |  |
| 8. | I use additional cleaning tools such as dental floss or interdental brushes.. |  |  |  |  |  |

*Questionnaire regarding the internal external locus using a 5-point scale

|  |  | No | | | Yes | | |
| --- | --- | --- | --- | --- | --- | --- | --- |
| 9. | Did you have access to  the GlobeSmile oral care video? |  | | |  | | |
| 10. | Have you heard about this oral care video from other residents? |  | | |  | | |
|  |  | Once | Twice | Three times | | Once per week | Daily |
| 11. | If so, how many times did you watch it? |  |  |  | |  |  |

*Questions concerning the accessibility and use of the oral health videos using a 2 and 5-point scale

**Supplementary Material S3: QR-code access card**

**Supplementary Figure 1:** Front and back of the QR-code access card providing multilingual access to the GlobeSmile oral health micro-learning video.


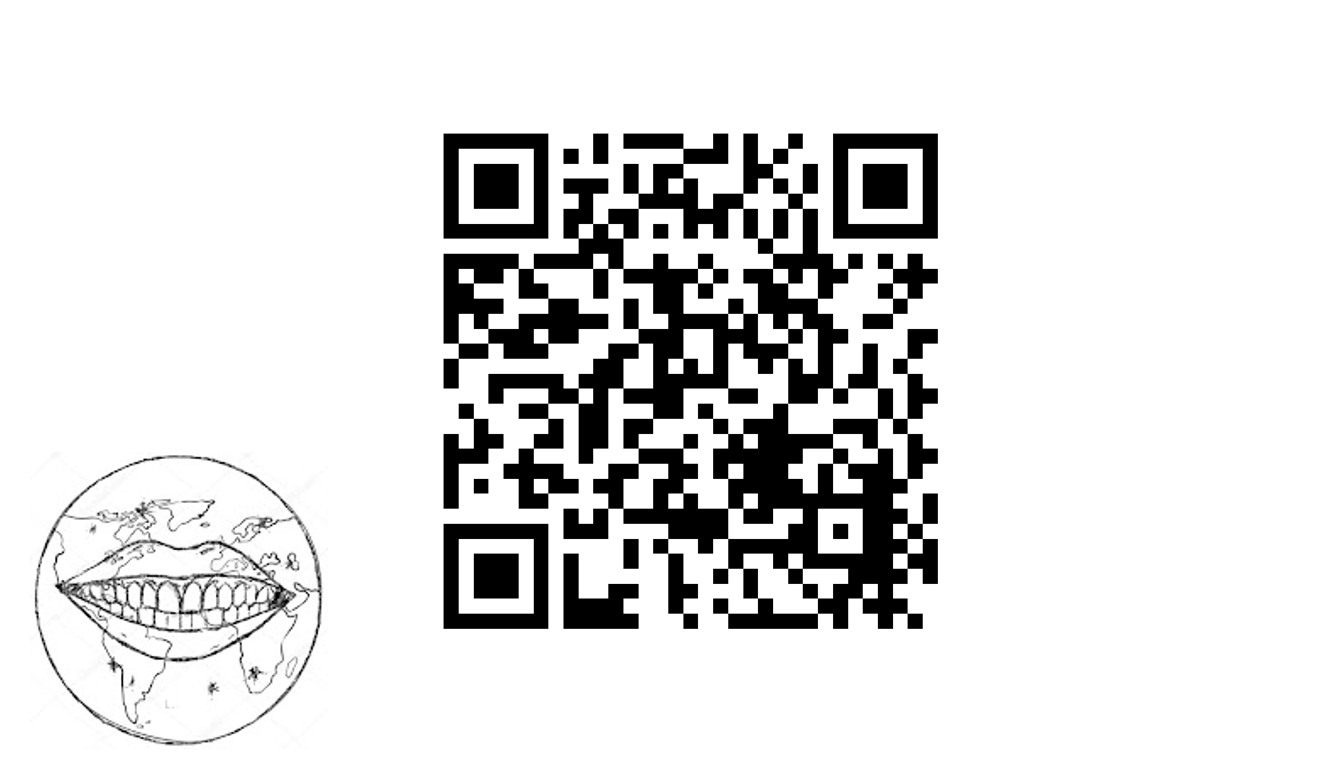

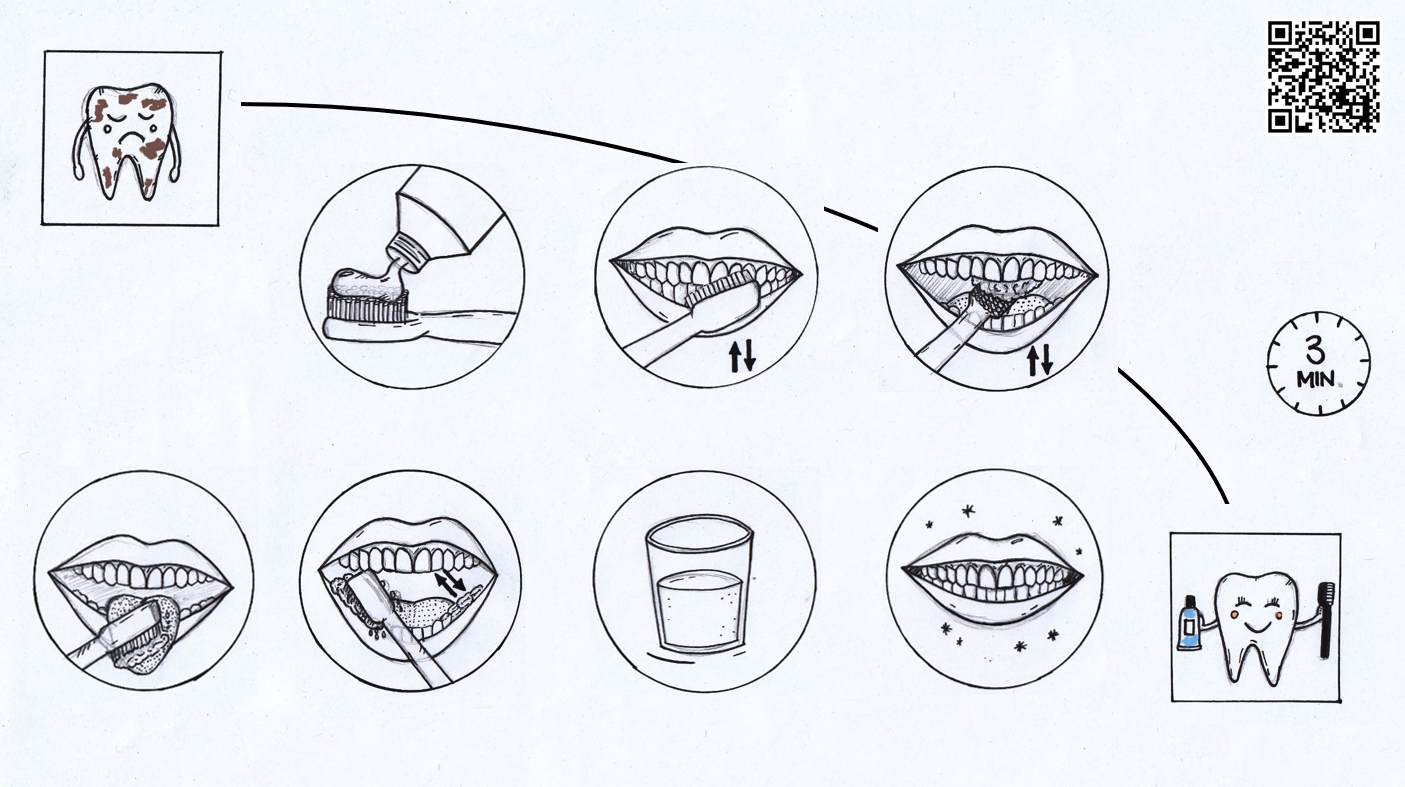

Supplement: Multimedia Appendix 1 [file jmir-v28-e95562-s001.docx]
