# Supplementary material for: A Multilingual Digital Microlearning Intervention for Oral Health in Refugee Shelters: Randomized Controlled Trial
Source: J Med Internet Res. 2026 Jun 15;28:e95562. doi: 10.2196/95562 (PMC13268633; doi:10.2196/95562)
Supplement: Checklist 1 [file jmir-v28-e95562-s002.pdf]

CONSORT-eHEALTH Checklist

Manuscript Title:
A Multilingual Digital Micro-Learning Intervention for Oral Health in Refugee Shelters: Randomized Controlled Trial

1. TITLE AND ABSTRACT

Item 1a – Identification as RCT in title

The title explicitly identifies the study as a randomized controlled trial.

Item 1b – Mode of delivery

The title specifies the intervention as a “digital micro-learning intervention.”

Item 1c – Target group

The target population (“refugee shelters”) is clearly stated.

Item 2a – Structured abstract

A structured abstract is provided, including Background, Objective, Methods, Results, and Conclusions.

Item 2b – Description of intervention

The abstract describes a brief (4-minute), multilingual animated oral health video, delivered via tablet with optional re-access through QR code.

Item 2c – Human involvement

Initial viewing was supervised in person; subsequent access was self-directed without additional support.

Item 2d – Recruitment and assessment

Participants were recruited and assessed in person in refugee shelters.

Item 2e – Usage data

Approximately 75% of participants reported re-viewing the intervention video at least once.

Item 2f – Outcomes

Primary outcomes: Plaque Index (PI), Gingival Index (GI).
Secondary outcomes: knowledge, attitudes, self-efficacy, and self-reported behaviours.

Item 2g – Conclusions

The abstract concludes that multilingual digital micro-learning may improve plaque control and selected oral health literacy outcomes.

2. INTRODUCTION

Item 3 – Problem description

Refugees face significant linguistic, structural, and informational barriers to preventive oral health care, contributing to health inequities.

Item 4 – Rationale for digital intervention

Digital, low-threshold, multilingual interventions offer a scalable strategy to address health information barriers in underserved populations.

Item 5 – Objectives

The study aimed to evaluate the effectiveness of a brief multilingual digital micro-learning intervention on clinical oral hygiene outcomes and oral health literacy–related outcomes.

3. METHODS

Study Design (Item 6a–b)

A two-arm, parallel-group randomized controlled trial with a 1:1 allocation ratio was conducted. No methodological changes occurred after trial initiation.

Participants (Item 7a–d)

Participants were adult residents (≥18 years) of refugee shelters who understood at least one intervention language and had ≥10 teeth per jaw.
No specific digital literacy requirements were imposed, as the intervention was designed to be low-threshold and accessible. Recruitment and data collection were conducted in person at two refugee shelters in Freiburg, Germany.

Intervention

Item 8a – Intervention name and description
The "GlobeSmile" intervention is a 4-minute animated multilingual oral health education video.

Item 8b – Development
The intervention was developed at the University Medical Center Freiburg using a participatory approach involving individuals with refugee backgrounds.

Item 8c – Version
A single finalized version of the intervention was used; no modifications occurred during the study.

Item 8d – Delivery mode
Participants viewed the video on a tablet at baseline and received a QR code for optional repeated access.

Item 8e – Setting
Hybrid delivery: in-person initial exposure and optional remote digital access.

Item 8f – Content
Content included toothbrushing technique, fluoride use, brushing duration, cleaning sequence, and tongue hygiene.

Item 8g – Languages
Arabic, Kurdish, Dari, Farsi, and Ukrainian.

Item 8h – Theoretical basis
The intervention is grounded in health literacy frameworks and observational learning principles.

Item 8i – User interaction
Passive video consumption with optional repeated viewing.

Item 8j – Prompts/reminders
No reminders or prompts were used.

Item 8k – Co-interventions
No additional interventions were provided.

Item 8l – Human support
Minimal support limited to initial tablet instruction.

Outcomes (Item 9a–d)

Primary outcomes were changes in Plaque Index (PI) and Gingival Index (GI).
Secondary outcomes included oral health knowledge, attitudes, perceived self-efficacy, and self-reported behaviours.
Intervention usage was assessed via self-reported re-viewing frequency.
Clinical outcomes were assessed through standardized examinations; questionnaires were administered in participants' native languages.

Sample Size (Item 10)

The sample size (n=86) was determined pragmatically based on the number of eligible residents rather than by formal power calculation.

Randomisation (Item 11a–c)

Participants were randomized in a 1:1 ratio using a simple randomization procedure.
Allocation was concealed using sequentially numbered, opaque, sealed envelopes prepared by an independent researcher.

Blinding (Item 12a–c)

Blinding of participants and outcome assessors was not feasible due to the nature of the intervention and setting. Data analysis was conducted blinded to group allocation.

Statistical Methods (Item 13a–c)

Within-group changes were analysed using Wilcoxon signed-rank tests.
Between-group differences were assessed using t-tests.
Multivariate linear regression analyses were conducted to explore predictors of outcome changes.
No significant missing data were observed among participants completing follow-up.

4. RESULTS

Item 14 – Participant flow

A total of 86 participants were randomized; 83 (97%) completed follow-up.

Item 15 – Baseline data

Baseline characteristics were comparable between groups.

Item 16 – Intervention use

Approximately 75% of participants reported re-viewing the video at least once.

Item 17 – Outcomes

A significantly greater reduction in Plaque Index was observed in the intervention group compared with controls.
No significant between-group difference was observed for Gingival Index.

Item 18 – Harms

No adverse events or unintended effects were reported.

5. DISCUSSION

Item 19 – Interpretation

The intervention improved plaque control, likely through improved brushing technique rather than substantial changes in behaviour frequency.

Item 20 – Limitations

Limitations include modest sample size, short follow-up duration, lack of blinding, potential contamination, and self-reported usage data.

Item 21 – Generalizability

Findings are generalizable to similar low-resource, multilingual, and high-mobility settings such as refugee shelters.

Item 22 – Mechanisms

Findings are consistent with health literacy frameworks, suggesting improvements in knowledge, self-efficacy, and behavioural skills.

6. OTHER INFORMATION

Item 23 – Trial registration

Registered in the German Clinical Trials Register (DRKS00032017).

Item 24 – Protocol availability

Study protocol and materials are available upon reasonable request.

Item 25 – Funding

No external funding was received.

Item 26 – Conflicts of interest

The authors declare no conflicts of interest
